# Assessment and Treatment of Negative Symptoms in Schizophrenia—A Regional Perspective

**DOI:** 10.3389/fpsyt.2021.820801

**Published:** 2022-02-04

**Authors:** Istvan Bitter, Pavel Mohr, Natalia Raspopova, Agata Szulc, Jerzy Samochowiec, Ioana Valentina Micluia, Oleg Skugarevsky, Róbert Herold, Alma Mihaljevic-Peles, Nino Okribelashvili, Jozef Dragašek, Virginija Adomaitiene, Elmars Rancans, Jana Chihai, Natalia Maruta, Nadja P. Marić, Vihra Milanova, Rok Tavčar, Sergey Mosolov

**Affiliations:** ^1^Department of Psychiatry and Psychotherapy, Semmelweis University, Budapest, Hungary; ^2^Clinical Center, National Institute of Mental Health, Klecany, Czechia; ^3^Third Faculty of Medicine, Charles University in Prague, Prague, Czechia; ^4^Department of Psychiatry, Narcology and Neurology of Kazakh-Russian Medical University, Almaty, Kazakhstan; ^5^Department of Psychiatry, Medical University of Warsaw, Warsaw, Poland; ^6^Department of Psychiatry, Pomeranian Medical University in Szczecin, Szczecin, Poland; ^7^Discipline of Psychiatry, Department of Neurosciences, University of Medicine and Pharmacy, Iuliu Haieganu, Cluj-Napoca, Romania; ^8^Department of Psychiatry & Medical Psychology, Belarusian State Medical University, Minsk, Belarus; ^9^Department of Psychiatry and Psychotherapy, Medical School, University of Pécs, Pécs, Hungary; ^10^Clinical Hospital Centre Zagreb and School of Medicine, University of Zagreb, Zagreb, Croatia; ^11^Department of Psychiatry and Medical Psychology, Tbilisi State University, Tbilisi, Georgia; ^12^First Department of Psychiatry, University of P. J. Safarik, Medical Faculty and University Hospital of L. Pasteur, Kosice, Slovakia; ^13^Department of Psychiatry, Lithuanian University of Health Sciences, Kaunas, Lithuania; ^14^Department of Psychiatry and Narcology, Riga Stradins University, Riga, Latvia; ^15^Medical Psychology and Narcology Department, State Medical and Pharmaceutical University Nicolae Testemitanu, Chisinau, Moldova; ^16^Department of Borderline Psychiatry, Institute of Neurology, Psychiatry and Narcology of the National Academy of Medical Science of Ukraine, Kharkiv, Ukraine; ^17^Faculty of Medicine University of Belgrade & Institute of Mental Health, Belgrade, Serbia; ^18^Psychiatric Clinic, University Hospital “Alexandrovska”, Sofia, Bulgaria; ^19^University Psychiatric Clinic Ljubljana and School of Medicine, University of Ljubljana, Ljubljana, Slovenia; ^20^Department for Therapy of Mental Disorders, Moscow Research Institute of Psychiatry, Moscow, Russia; ^21^Department of Psychiatry, Russian Medical Academy of Continuous Professional Education, Moscow, Russia

**Keywords:** negative symptoms, schizophrenia, assessment, treatment, review, personality, Central and Eastern Europe

## Abstract

Clinicians and researchers consider that there are a variety of symptoms that constitute negative symptoms in schizophrenia, and they may use different definitions for the same symptoms. These differences are also reflected in a variety of negative symptom rating scales. Both research and clinical work are negatively affected by the lack of consensus regarding the symptoms that constitute negative symptoms in schizophrenia. Leading research groups have investigated ways to reduce heterogeneity in the domain of negative symptoms in schizophrenia; however, little attention has been paid to regional differences in the concepts of negative symptoms in schizophrenia. The objective of this review was to collect and summarize information about the assessment and treatment of negative symptoms of schizophrenia in Central and Eastern Europe (CEE). Nineteen experts from 17 countries in CEE participated in this project. The participants collected information about their countries, including the following: (1) the most important publications about negative symptoms in schizophrenia (irrespective of the time of their publication); (2) the most frequently used negative symptom of schizophrenia in clinical practice; (3) definitions of frequently used negative symptoms; and (4) treatment of negative symptoms in schizophrenia. The participating experts/countries most frequently reported the following five negative symptoms: avolition, blunted affect, alogia, asociality, and anhedonia. Several experts also considered other symptoms as belonging to the negative symptom domain, such as a decrease in energy level and changes in personality. The importance of evaluating the long-term course and the relationship between negative symptoms and other symptom domains was also noted. No noticeable differences were reported in the treatment of negative symptoms compared to currently published guidelines and algorithms. The most frequently reported negative symptoms included those defined by the NIMH-MATRICS consensus statement on negative symptoms and recently endorsed in a guidance paper of the European Psychiatric Association. The main differences in the concepts, names, and definitions of primary negative symptoms, especially those related to personality changes, and to the evaluation of the long-term course and relationship between different symptom domains in CEE compared to the current English language literature deserve the attention of psychiatrists and other professionals in this field.

## Introduction

Clinicians and researchers consider a variety of symptoms as negative symptoms of schizophrenia and may use different definitions for the same symptom (1). This ambiguity is also reflected by a variety of negative symptom rating scales, which include a range of different negative symptoms, sometimes with the same name but with different definitions (2). Both research and clinical work are negatively affected by the lack of consensus regarding negative symptoms. Expert groups addressed ways to improve the definitions of negative symptoms in schizophrenia in order to improve their assessment and treatment (2–4); however, little attention has been paid to regional—including cultural—differences in the clinical approaches of different schools of psychiatry.

Examples of geographical/regional differences in psychiatry that have been previously addressed include the following: in the context of large regional variability in the time to all-cause discontinuation of antipsychotic treatment of schizophrenia (5, 6); or “the lack of uniformity in the definition of treatment resistant depression (TRD) within the Asia-Pacific (APAC) region,” which “may have implications for patient management” (7). A review on mental health care for people with severe mental illnesses in Central and Eastern Europe (CEE) resulted in a large “review on mental health systems in the former Eastern Bloc”; however, the focus of this work was on the structure and functioning of mental health care in post-communist countries rather than research, medical education, and training (8). The authors of a literature review on the “Epidemiology and Treatment Guidelines of Negative Symptoms in Schizophrenia in Central and Eastern Europe…” (9) concluded the following: “Despite the extensive search, we were unable to find relevant data in all areas of interest.” A similar conclusion was made by the authors of a previously published paper about psychiatry in nine CEE countries: “There is a great tradition of psychiatry in the region; however, the scientific output and number of psychiatric publications in international peer-reviewed journals is considerably low.” (10). However, in another study focusing on patients with a diagnosis of schizophrenia in CEE, the authors reported that the information provided by selected experts was useful (11), which is similar to the conclusion in the paper by Winkler et al. (8).

The discussion of the history of psychiatry and the heterogeneity of higher education and research systems in CEE countries is far beyond the scope of this paper; however, it is important to emphasize that these countries are rather heterogeneous. Indeed, CEE countries are even more heterogeneous than countries in Western Europe. Some CEE countries were part of the Habsburg Empire; for example, Czechia, Croatia, Hungary, Slovakia, Slovenia, parts of Poland, Ukraine, and Romania, and others have been in close contact with Russia and were included in the Soviet Union. Most countries in the region were significantly influenced by German psychiatry until the 1980s, while other countries (especially the former republics of the Soviet Union) had ties to Russian psychiatry. Considering the representation of psychiatry as a medical discipline in each respective country, we find substantial differences in the history of psychiatry. For example, Tbilisi State University was founded in 1918 (https://en.wikipedia.org/wiki/Tbilisi_State_University), which contributed to the development of the discipline of psychiatry in the native language in Georgia (12). In contrast, Kraepelin was the Professor of Psychiatry at the University of Tartu (earlier: Dorpat) in Estonia between 1886 and 1891. Meanwhile, the Charles University in Prague was founded in 1348. “As a date of the very beginning of the Psychiatric Clinic of the Czech University, the November 19th, 1886, is considered.” Before this date, the language of teaching in Prague was German and the same applied to Dorpat. The University in Vilnius (Lithuania) was founded in 1579 and has been a leading university in Europe. The names of the two founding fathers of Russian psychiatry, Korsakoff (1854-1900) from Moscow (https://link.springer.com/referenceworkentry/10.1007%2F978-0-387-79948-3_631) and Bekhterev (1857-1927) from St. Petersburg (https://en.wikipedia.org/wiki/Vladimir_Bekhterev), are well-known worldwide. After the major political changes in the CEE region in the 1990s, English language publications and US psychiatry had an overwhelming influence on psychiatry. Several countries from the CEE region became EU member states, and psychiatrists also participated in EU-funded research and educational projects.

There is a detectable increase in interest in the negative symptoms of schizophrenia. Based on data from Google Scholar, 44% of papers on schizophrenia published in 2000 and 73% published in 2020 (38,900/53,200 hits) refer to negative symptoms (12,100/27,600 hits) (the search was performed on October 16, 2021, and the search terms were “schizophrenia negative symptoms” and “schizophrenia,” respectively). During professional meetings, the authors of this paper had the opportunity to discuss the development of research on negative symptoms in schizophrenia and concluded that some current issues in this field could be more efficiently addressed with contributions from experts working in CEE. These contributions may pose some challenges, since some contributions do not simply increase the amount of current information about negative symptoms in schizophrenia, but also raise questions about the usefulness of current phenotyping in schizophrenia for much needed basic research, including drug discovery and development for the treatment of this disease. Examples include long-term evaluation of negative symptoms and personality changes during the course of schizophrenia.

The objective of this scoping review was to summarize the selected literature and expert opinions on negative symptoms in schizophrenia from CEE countries.

## Methods

Nineteen experts (the authors of this paper) from 17 countries participated in this project. We used the World Health Organization's definition of Europe, which includes some Asian countries. The selection of countries also reflects the availability of interested experts in the region. The 17 countries are Belarus, Bulgaria, Croatia, Czech Republic, Georgia, Hungary, Kazakhstan, Latvia, Lithuania, Moldova, Poland, Romania, Russia, Serbia, Slovakia, Slovenia, and Ukraine.

The coordinator of the project (IB) distributed a questionnaire and collected additional information from the project participants, mainly via email communication. The questionnaire included the following questions and requests with additional instructions.

- Request for the identification of the most important publications on negative symptoms of schizophrenia in the participating countries, irrespective of the time of their publication.
- Request for a list of the negative symptoms of schizophrenia used in clinical practice in the participating countries ranked by their “popularity” and endorsed by academia (e.g., textbooks). We collected all symptoms that were considered negative symptoms in the participating countries, irrespective of the current views on negative symptoms.
- Request for the description of the definitions of the listed negative symptoms (e.g., “How are they defined in well-accepted textbooks in your language/country?”).
- The following questions were included in the questionnaire: “Are there recommendations in the participating country for the treatment of negative symptoms in schizophrenia? If yes, please refer to (short summary).” If no recommendation existed, participants were asked to summarize the clinical practice for the treatment of negative symptoms in their countries. Based on the participants' responses regarding lack of or limited availability of psychosocial interventions for negative symptoms in schizophrenia, the question was changed and included only pharmacological treatment.

## Results

### Evaluation

Online Supplementary 1 includes a list of selected references provided by participating experts. The literature provided by the experts addressed the assessment more than the treatment of negative symptoms. Many important contributions are available only in the local languages. Russian is also used in some countries that were republics of the Soviet Union. A few of the provided references were from outside the country of the responding expert, which illustrates the importance of some authors and/or schools of psychiatry in a specific country; they are either translated into the language used in the country or published in English or Russian.

The literature collected by the participants of this regional project shows that negative symptoms are considered important, and the selected literature and expert opinions reflect high-level interest in the negative symptoms of schizophrenia in CEE countries. In the majority of CEE, the terminology of negative symptoms is dominated by English, as summarized in the currently published European guidance paper on the evaluation of negative symptoms in schizophrenia (2) however, traditional German psychopathology [e.g., the work of Huber (13, 14)] has a significant influence in the region, and Russian schools have a strong influence not only in Russia, but also in some other countries, especially in former republics of the Soviet Union (15, 16). Table 1 includes the names and frequencies of negative symptoms listed by experts from 17 countries.

**Table 1:** List of the negative symptoms used in 17 countries across CEE.

**Symptom**	**Times mentioned**	**Country/countries endorsed**
**Avolition** Reduction of volitive action, Abulia Abulia Abulia-apathy^*^ Reduced volitional activity	16	Croatia, Czech Republic, Georgia, Hungary, Latvia, Lithuania, Moldova, Poland, Romania, Russia, Serbia Slovakia Kazakhstan Belarus Ukraine Bulgaria
**Blunted affect, flat affect, Affective flattening / blunting** Impoverishment of emotional reactions and blunted affect, Affective blunting, and poverty of emotional expression Lack of emotional expression Flattening of affect (apathy) Blunted or inappropriate affect	16	Croatia, Czech Republic, Hungary, Kazakhstan, Latvia, Lithuania, Moldova, Poland, Romania, Slovakia, Ukraine, Russia Serbia Belarus Bulgaria Slovenia
**Asociality** Lack of social interest Social withdrawal Social withdrawal Limitation of social contacts Anhedonia-asociality Social withdrawal	14	Czech Republic, Georgia, Hungary, Kazakhstan, Lithuania, Poland, Serbia, Ukraine Croatia Romania Russia Slovakia Bulgaria Slovenia
**Alogia** Poverty of quantity or content of speech No spontaneity/fluency in discussion (alogy) Poverty of verbal expression	14	Czech Republic, Georgia, Hungary, Kazakhstan, Russia, Lithuania, Moldova, Romania, Serbia, Ukraine, Slovenia Latvia Poland Slovakia, Russia
**Anhedonia** Anhedonia-asociality	11	Croatia, Czech Republic, Georgia, Hungary, Kazakhstan, Lithuania, Moldova, Romania, Serbia, Ukraine, Bulgaria
**Apathy** Abulia-apathy^*^ Flattening of affect (apathy) Apathy, Abulia, Hypobulia	6	Georgia, Russia, Slovakia Ukraine Bulgaria Slovenia
**Autism**	4	Croatia, Moldova, Kazakhstan, Russia
**Personality changes**	3	Kazakhstan, Russia, Ukraine
**Emotional withdrawal**	2	Croatia Poland
Psychomotor slowing Motor retardation	2	Latvia Poland
Passivity, Indifference	2	Latvia, Belarus
Lack of initiative	2	Latvia, Belarus
Reduction of energy capacity/reduced mental activity, Asthenia	2	Russia, Kazakhstan
Poor rapport, poor contact with others	2	Croatia, Poland
Poor social performance Poor social functioning	2	Latvia, Slovenia
Underactivity	1	Latvia
Poor self-care	1	Latvia
Deficit syndrome	1	Lithuania
Poor non-verbal communication	1	Latvia
Deterioration/reduction of intellectual activity	1	Russia
Indecisiveness	1	Slovakia
Narrowing of the circle of interests	1	Belarus

* *The item “abulia-apathy” appears both as “abulia” associated with the item “avolition” and as “apathy” associated with the item “apathy”*.

The following symptoms were collected by Raspopova from Kazakhstan: “emotional decline, socially withdrawn and passive, gross personality changes, narrowing of the range of interests, increasing autism, slowly increasing autism, slight decrease in energy potential, violation of logical harmony and of the purposefulness of thinking; disorganization of psychic activity; a significant decrease in interests and activity characteristic for a given person; emotional impoverishment; increasing social withdrawal (autism); various disorders of thinking and behavior.” Raspopova et al. published a study guide in Russian focusing on negative symptoms and provided a detailed review on cariprazine (17). The following terms were included in a 1968 dissertation by Chiladze (1968; see Online Supplementary 1): (1) the “loosening up” of concepts; (2) poverty of imagination/fantasy; (3) the flatness of spiritual feelings; (4) emotional alienation; (5) indifferent/“indisciplined.” These two examples uncover the results of old research in the field and show the wealth of terms used to describe the devastating effects of schizophrenia.

The most noticeable difference in the current descriptions of negative symptoms in the English language literature is the inclusion of “personality changes (“personality shift”)” in the negative symptom domain in some countries (e.g., Russia, Ukraine, and Kazakhstan) and the proposed analysis of the long-term course and relationship of negative symptoms to positive and other symptom domains during the course of schizophrenia (16, 18).

Based on the selected publications from the region and on the written reports from the authors of this review, we found that in routine clinical practice, negative symptoms are not stratified by the doctors in most countries into “primary” and “secondary” symptoms. Cognitive symptoms are often not differentiated; however, Capatina and Miclutia (19) published a study demonstrating the absence of a relationship between cognitive and negative factors when using the Positive and Negative Syndrome Scale (PANSS) scale. Nevertheless, when investigating the relationship of the PANSS cognitive factor with the negative symptoms evaluated using the Negative Symptom Assessment (NSA-16) scale, it was shown that there was a significant association between cognition and motor retardation. They concluded that negative symptoms represent a separate treatment target. The same group identified studies on the stability of negative symptoms over 1 year (20), as well as secondary negative symptoms. In their review, they reported the following. “Factorial analyses showed that secondary negative symptoms encompass the same domains as primary negative symptoms: avolition/apathy and diminished expression, but it is not yet clear, and evidence are sparse regarding how specific causes of secondary negative symptoms are related which negative symptom domain. Although recent research has defined the main causes of secondary negative symptoms, evidence-based treatment recommendations remain scattered.”

Based on a survey of the Russian Association of Psychiatry, including 807 psychiatrists representing 78 regions of Russia, 35% of the respondents supported the proposal that negative symptoms should be defined and considered obligatory for the diagnosis of schizophrenia in the diagnostic systems, and the respondents estimated that the specificity of “emotional-volitional reduction” for schizophrenia was 72.1% (SD = 19.6; *n* = 685) (21).

None of the participants in this study reported ethnic or transcultural diversities in the description of negative symptoms in patients with schizophrenia, either in scientific research or in clinical practice.

### Pharmacological Treatment

All experts reported that the current literature on the treatment of negative symptoms is available in their countries. Approaches to the treatment of negative symptoms in schizophrenia in the CEE [e.g., (22–24)] are in line with the current literature, including suggested algorithms, guidelines, and reviews about the treatment of negative symptoms (3, 25, 26). In some countries (e.g., Georgia and Romania), cariprazine was unavailable at the time of writing this manuscript. A number of countries (e.g., Czechia, Kazakhstan, Russia, and Slovakia) incorporated the treatment algorithm for negative symptoms in schizophrenia suggested by Cerveri et al. (2, 24) in their new guidelines or in other publications, which reflects the current progress and limitations in the field. The authors emphasize the need for further research on the treatment of primary or predominant and persistent negative symptoms. Their recommendation is to use cariprazine as a first-line drug for the treatment of negative symptoms in schizophrenia; amisulpride is a second-generation antipsychotic with a partial agonist effect on D2/D3 dopamine receptors and with variable levels of evidence (3). The third-line options include other second-generation antipsychotics (SGAs), specifically olanzapine and quetiapine, and the fourth option is the addition of antidepressants. The Czech guidelines also cautiously suggest high-frequency, repetitive transcranial magnetic stimulation (rTMS) administered over the left dorsolateral prefrontal cortex (DLPFC Czechia, Croatia, and Russia have reported the use of rTMS for negative symptoms in patients with schizophrenia with variable outcomes (27–30).

## Discussion

### Evaluation

The majority of the participating experts/countries endorsed the five main symptoms as conceptualized by the NIMH-MATRICS consensus statement on negative symptoms: avolition, blunted affect, alogia, asociality, and anhedonia (4). Avolition and blunted affect were the most frequently endorsed symptoms in the CEE study. Research of the psychopathology of “will” has long been a tradition both in Germany and Russia, which has been addressed by Mosolov and Yaltonskaya (in press) (12). The severity (from mild inhibition of will to lack of will), components (drive, imagination, decision making, etc.), and temporal phases of the disturbances of will are present in different works about negative symptoms from CEE; however, those details [see for example (31)] are not included in currently used rating scales for negative symptoms (2). A current treatment study suggests that decoupling the influence of motivational processes from other negative symptom domains is essential for producing global improvements. The search for pathophysiological mechanisms and targeted treatment development should focus on avolition, with the expectation of improvement in the entire constellation of negative symptoms if avolition is effectively treated (32). Considering this finding and the accumulated knowledge about the disturbances of “will” (31), a detailed analysis of the components of avolition and their relationship to constructs, such as drive, motivation, decision making, asthenia, ambivalence, etc., may help make significant advances in this field. For example, we have witnessed how the distinction between anticipatory and consummatory pleasure changed our thinking about anhedonia in schizophrenia (33). Additional symptoms were also named in CEE; for example, negative symptoms from Russia, Kazakhstan, and Ukraine included “decrease in energy potential” and “personality changes,” where “decrease in energy potential” was defined as the initial core symptom in the hierarchy of negative symptoms.

The complex relationship between schizophrenia and personality disorders has been an important topic in psychopathology, with a focus on personality disorders during the premorbid and prodromal phases of schizophrenia and in the differential diagnosis of schizophrenia (34). The Russian School of Psychopathology includes personality changes in the hierarchy of negative symptoms associated with poor outcomes in patients with schizophrenia. Personality changes as negative symptoms have also been reported by Ukrainian and Kazakh experts. They refer to a hierarchy of negative symptoms by the level of their severity and report 10 levels, which include several personality-related negative symptoms.

The Russian school also emphasizes the “long-term course of negative symptoms and their relationship” between symptom domains in the course of schizophrenia; for example, it differentiates between a “synchronized” and “desynchronized” course of positive and negative symptoms (18). In the case of a “synchronized” relationship between positive and negative symptoms, the secondary nature of the increase in severity of negative symptoms can be hypothesized, while in the case of a “desynchronized” course, the increase in the severity of negative symptoms is not associated with an increase in positive symptoms. The 11th edition of the International Statistical Classification of Diseases and Related Health Problems (35) includes the following “Symptom Specifiers” for the cross-sectional characterization of the symptomatology in schizophrenia: “Positive symptoms,” “Negative symptoms,” “Depressive symptoms,” “Manic symptoms,” “Psychomotor symptoms,” and “Cognitive impairments.” These specifiers should characterize the symptom status of a patient only for 1 week and not over longer periods of time; thus, the length of time of the presence or absence of a specifier is a major difference between the approach of ICD-11 and of the representatives of Russian psychopathology (18). The requirement of predominant negative symptoms for a long period by the European Medicines Agency results in the exclusion of a group of patients with primary negative symptoms, which is one of the reasons that the concept of predominant negative symptoms as an inclusion criterion in clinical trials has been challenged (1, 2). There is a well-defined need for more research into the primary symptoms of schizophrenia (36).

### Pharmacological Treatment

In contrast to the evaluation of negative symptoms in schizophrenia, we found no differences between the current evidence-based literature (3, 26, 37) and the opinions of experts and papers published on this topic by experts from CEE countries. This finding is not surprising, considering that very few drugs are available worldwide that have any evidence of efficacy for the treatment of negative symptoms of schizophrenia. The third and fourth switches of medication indicate treatment resistance. The concept of treatment-resistant schizophrenia is broader than resistance to the treatment of negative symptoms; however, it also includes negative symptoms. In addition, the evidence base is quite low at the 3rd or 4th step of the proposed treatment algorithms for primary negative symptoms; for example, the recommendation of quetiapine is based on a small study with 44 patients comparing quetiapine to risperidone (23).

Our study highlights the need for more intensive collaborative research and dialogue between researchers in different parts of the world.

## Limitations

The 17 countries included in the study cover a large proportion of, but not all, countries in CEE. Data collection was based on the opinion of participating experts; however, the selected experts had a demonstrable track record (oral presentations during national and international meetings, and published books, papers, and abstracts about or closely related to the topic of negative symptoms in schizophrenia).

## Conclusions

Our review reports the most important information regarding the evaluation and treatment of negative symptoms in a large geographic region. The region has rich diversity in the form of different languages, traditions, and current trends in psychiatry. The main differences in the concepts, names, and definitions of primary negative symptoms in CEE compared to the current English language literature deserve the attention of psychiatrists and other interested professionals, such as the inclusion of personality changes in the negative symptom domain or the importance of considering the long-term course of negative symptoms in schizophrenia.

## Author Contributions

IB: concept, coordination of the project that resulted in this manuscript, and drafting the manuscript of this review. All authors reviewed and approved the concept, collected data on negative symptoms in their countries (on the use, definition, evaluation, and pharmacological treatment of negative symptoms and country-specific references to these topics), contributed with their summaries to the draft manuscript, reviewed the draft manuscript, reviewed, and approved the final manuscript.

## Funding

The fee for language editing and the open access publication fee were covered by unrestricted support from Gedeon Richter. IB's work was partly supported by the Hungarian Brain Research Program (2017-1.2.1-NKP-2017-0002). RH's work was supported by the National Brain Research Program [Grant No. NAP KTIA NAP-A-II/12(2017-2021)] and The National Excellence programme (2019-2021, FIKP II.).

## Conflict of Interest

In the past 5 years, IB received honoraria or consultation fees outside of this work from Angelini, Eli Lilly, Gedeon Richter, Hikma Pharmaceuticals, Janssen/Janssen Cilag, Lundbeck, Medichem Pharmaceuticals, Inc. by Unilab, Philippines, Mitsubishi Tanabe Pharma Singapore, and Sun Pharma. He received royalties from the Oxford University Press. In the past 5 years, VA received honoraria or consultation fees from Janssen/Janssen Cilag. JS received honoraria as a member of the Speaker Bureau of Gedeon Richter. In the past 5 years, RT received speaker's honoraria from Angelini, Gedeon Richter, Janssen, Krka, Lek, Lundbeck, Mylan, Promed, Servier, Teva. In the past 5 years, AM-P received honoraria as speaker from Gedeon Richter, Janssen, Lundbeck, Pliva-Teva. PM has been a consultant and received honoraria and/or speaker fees from Angelini Pharma. Janssen-Cilag, Gedeon Richter, Lundbeck, and Viatris Mylan. In the past 5 years, NM received honoraria or consultation fees from Gedeon Richter, Maylan, Actavis-Teva and Pfizer. AS received honoraria or consultation fees from Gedeon Richter, Janssen Poland, and Angelini, Bausch Poland. In the past 5 years, RH received honoraria or consultation fees from Egis, Eli Lilly, Gedeon Richter, Janssen, Krka, Lundbeck, Mylan-Viatris, Servier, and Teva. In the past 5 years, ER received research grants from Gedeon Richter and Lundbeck, speaker honoraria, and is a member of advisory panels for Abbvie, Gedeon Richter, Grindex, Janssen Cilag, Lundbeck, Servier, and Zentiva. He has been the principal investigator in clinical trials for Lundbeck, Janssen Cilag, and Sunovion. In the past 5 years, SM received honoraria or consultation fees from Angelini, Gedeon Richter, Janssen, Lundbeck, Abbott, Grindex, and Servier. In the past 5 years, IM received honoraria as speaker from Angelini, Gedeon Richter, Janssen J&J, Lundbeck, Plantextract, and Terapia. NM has received in the past 5 years honoraria or consultation fees from Gedeon Richter, Sanofi, Acino, OlainFarm, Ever-pharma, Dileo Farma. NO, as a speaker, has received honoraria from Abbott, Abdi Ibrahim, Acino, Angelini, Egis, Gedeon Richter, Grindex, Novartis, Sanofi Aventis, Servier in the past 5 years. JD has received in the past 5 years honoraria or consultation fees from Angelini, Eli Lilly, Gedeon Richter, Janssen, Krka, Lundbeck, Sandoz. VM has received in the past 5 years honoraria or consultation fees from Angelini, Gedeon Richter, Janssen/Janssen Cilag, Medochemie, Lundbeck. The remaining authors declare that the research was conducted in the absence of any commercial or financial relationships that could be construed as a potential conflict of interest. The handling editor declared a past co-authorship with one of the authors IB.

## Publisher's Note

All claims expressed in this article are solely those of the authors and do not necessarily represent those of their affiliated organizations, or those of the publisher, the editors and the reviewers. Any product that may be evaluated in this article, or claim that may be made by its manufacturer, is not guaranteed or endorsed by the publisher.
