# Supplementary material for: Assessment and Treatment of Negative Symptoms in Schizophrenia—A Regional Perspective
Source: Front Psychiatry. 2022 Feb 4;12:820801. doi: 10.3389/fpsyt.2021.820801 (PMC8855151; doi:10.3389/fpsyt.2021.820801)
Supplement: Supplementary file 1 [file Data_Sheet_1.PDF]

Online Supplement 1. List of selected publications on negative symptoms in schizophrenia from 17 CEE countries

Bulgaria

Haralanov S, Haralanova E. Negativni simptomi pri shizofrenia: osobenosti na mehanizmite I vazmojnostite za lechenie. (Negative and pseudonegative symptoms in schizophrenia: features of mechanisms and treatment perspective). GPNews (2020) 21(1): 5-9. https://gpnews.bg/%D1%81%D0%B8%D0%BC%D0%BF%D1%82%D0%BE%D0%BC%D0%B8%D0%BF%D1%80%D0%B8-%D1%88%D0%B8%D0%B7%D0%BE%D1%84%D1%80%D0%B5%D0%BD%D0%B8%D1%8F

Haralanov S, Haralanova E. Negativni simptomi pri shizofrenia: mehanizmi I vazmojnosti za lechenie. (Negative symptoms in schizophrenia: mechanisms and treatment). (Pro Medic) (2019) 1(1): 46-54.

Haralanov S, Haralanova E, Shkodrova D. Vazmojnosti za lechenie na negativni simptomi pri shizofrenia. (Treatment of negative symptoms in schizophrenia). MedInfo (2019) 11: 56-61. https://www.medinfo.bg/spisanie/2019/11/statii/vyzmojnosti-za-lechenie-na-negativni-simptomi-pri-shizofrenija-3015

Tolev T, Tolev A, Tranov V, Nikolova M, Grozeva P, Terzieva D, Drebova K, Terzieva A, Batzeva T, Patricheva R. Simptomi I deficit v dekodirane na liceizraz na taga pri pacienti s shizofrenia. (Clinical symptoms and deficit in decoding facial expressions of sadness in patients with schizophrenia). Bulgarian Journal of Psychiatry, (2016) 1 (1):68-81. http://d2lwoed6slg2cc.cloudfront.net/uploads%2FContent%2FStatuses%2FBgJ_Psych_2016_Vol_1(1)_final.pdf

Masaldjiev R, Akabalieva K, Milanova V, Tolev T, Grozeva P, Pashova M. Negativna simptomatika I socialno funkcionirane pri paranoidna shizofrenia. (Negative symptoms and social functioning in paranoid schizophrenia). Folia Psychiatrica (2011) 2(3):28-32.

Belarus

Kozhanova I, Romanova I, Sachek M. Pharmacoeconomic evaluation of the use of the drug Reagila (cariprazine) for the treatment of patients with schizophrenia with negative symptoms. Psychiatry psychotherapy and clinical psychology (2020) 11(3).

Skugarevsky O. The Concept of Negative Symptoms of Mental Disorders: Time to Recharge. Psychiatry psychotherapy and clinical psychology (2020) 11(2). doi: 10.34883/PI.2020.11.2.009

Croatia

Madzarac Z, Tudor L, Sagud M, Nedic Erjavec G, Mihaljevic Peles A, Pivac N. The Associations between COMT and MAO-B Genetic Variants with Negative Symptoms in Patients with Schizophrenia. Curr Issues Mol Biol (2021) 43(2):618-36. doi: 10.3390/cimb43020045

Mihaljevic-Peles A, Janovic MB, Strucic A, Sagud M, Hanzek MS, Zivkovic M, et al. Electroconvulsive Theraphy - General Considerations and Experience in Croatia Psychiatria Danubina (2018). p. S10-909.

Mihaljevic-Peles A, Bajs Janovic M, Sagud M, Zivkovic M, Janovic S, Jevtovic S. Cognitive deficit in schizophrenia: an overview. Psychiatr Danub (2019) 31(Suppl 2):139-42.

Sagud M, Tudor L, Simunic L, Jezernik D, Madzarac Z, Jaksic N, et al. Physical and social anhedonia are associated with suicidality in major depression, but not in schizophrenia. Suicide Life Threat Behav (2021) 51(3):446-54. doi: 10.1111/sltb.12724

Czech Republic

Assessing the effects of atypical antipsychotics on negative symptoms. Collaborative Working Group on Clinical Trial Evaluations. J Clin Psychiatry (1998) 59 Suppl 12:28-34.

Andreasen NC. Negative symptoms in schizophrenia. Definition and reliability. Arch Gen Psychiatry (1982) 39(7):784-8. doi: 10.1001/archpsyc.1982.04290070020005

Cerveri G, Gesi C, Mencacci C. Pharmacological treatment of negative symptoms in schizophrenia: update and proposal of a clinical algorithm. Neuropsychiatr Dis Treat (2019) 15:1525-35. doi: 10.2147/NDT.S201726

Crow TJ. Positive and negative schizophrenic symptoms and the role of dopamine. Br J Psychiatry (1980) 137:383-6. doi: 10.1136/bmj.280.6207.66

Masopust J, Mohr P, Anders M. Treatment of negative symptoms in schizophrenia. Psychiatrie (2018) 22(3):151-60.

Masopust J, Kopeček M, Protopopová D. Stabilizing and maintenance treatment of schizophrenia. Guidelines of psychiatric care 2018 (update 2020). Psychiatrie (2020).

Masopust J, Mohr P, Kopeček M. Antipsychotics in treatment of predominant negative symptoms in schizophrenia: an update of guidelines. Psychiatrie (2020) 24(1):40-3.

Masopust J, Mohr P, Kopeček M. Cariprazine. An antipsychotic for (not only) treatment of negative symptoms of schizophrenia. Praha: Galén (2021). 159 p.

Meltzer HY. What's atypical about atypical antipsychotic drugs? Curr Opin Pharmacol (2004) 4(1):53-7. doi: 10.1016/j.coph.2003.09.010

Mohr P. Deficit syndrome of schizophrenia: a review. Psychiatrie (1998) 2:94-102.

Novak T, Horacek J, Mohr P, Kopecek M, Skrdlantova L, Klirova M, et al. The double-blind sham-controlled study of high-frequency rTMS (20 Hz) for negative symptoms in schizophrenia: negative results. Neuro Endocrinol Lett (2006) 27(1-2):209-13.

Leucht S, Corves C, Arbter D, Engel RR, Li C, Davis JM. Second-generation versus first-generation antipsychotic drugs for schizophrenia: a meta-analysis. Lancet (2009) 373(9657):31-41. doi: 10.1016/S0140-6736(08)61764-X

Lewander T. Neuroleptics and the neuroleptic-induced deficit syndrome. Acta Psychiatr Scand Suppl (1994) 380:8-13. doi: 10.1111/j.1600-0447.1994.tb05825.x

Libiger J. Negative symptoms: resistant core of schizophrenia. Psychiatrie (2018) 22:39-46.

Riedel M, Muller N, Strassnig M, Spellmann I, Engel RR, Musil R, et al. Quetiapine has equivalent efficacy and superior tolerability to risperidone in the treatment of schizophrenia with predominantly negative symptoms. Eur Arch Psychiatry Clin Neurosci (2005) 255(6):432-7. doi: 10.1007/s00406-005-0622-6

Prikryl R, Kasparek T, Skotakova S, Ustohal L, Kucerova H, Ceskova E. Treatment of negative symptoms of schizophrenia using repetitive transcranial magnetic stimulation in a double-blind, randomized controlled study. Schizophr Res (2007) 95(1-3):151-7. doi: 10.1016/j.schres.2007.06.019

Prikryl R, Skotakova S, Kasparek T, Ceskova E, Kucerova H, Ustohal L. Influencing negative symptoms of schizophrenia with repetitive transcranial magnetic stimulation: a case study. Acta Neuropsychiatr (2007) 19(1):53-5. doi: 10.1111/j.1601-5215.2006.00166.x

Přikryl R, Kučerová H. Negative symptoms of schizophrenia. Česká a slovenská psychiatrie (2008) 104(7):350-7.

Prikryl R. Repetitive transcranial magnetic stimulation and treatment of negative symptoms of schizophrenia. Neuro Endocrinol Lett (2011) 32(2):121-6.

Prikryl R, Mikl M, Prikrylova Kucerova H, Ustohal L, Kasparek T, Marecek R, et al. Does repetitive transcranial magnetic stimulation have a positive effect on working memory and neuronal activation in treatment of negative symptoms of schizophrenia? Neuro Endocrinol Lett (2012) 33(1):90-7.

Prikryl R, Kucerova HP. Can repetitive transcranial magnetic stimulation be considered effective treatment option for negative symptoms of schizophrenia? J ECT (2013) 29(1):67-74. doi: 10.1097/YCT.0b013e318270295f

Prikryl R, Ustohal L, Prikrylova Kucerova H, Kasparek T, Venclikova S, Vrzalova M, et al. A detailed analysis of the effect of repetitive transcranial magnetic stimulation on negative symptoms of schizophrenia: a double-blind trial. Schizophr Res (2013) 149(1-3):167-73. doi: 10.1016/j.schres.2013.06.015

Georgia

Ministry of Internally Displaced Persons From the Occupied Territories L, Health and Social Affairs of Georgia. Treatment and Management of Schizophrenia in Adults. (2013). p. https://www.moh.gov.ge/ka/guidelines/.

Chiladze Ts. A. Final states of the paranoid form of schizophrenia (Clinical and laboratory research). (Konechnyye sostoyaniya paranoidnoy formy shizofrenii (Kliniko-laboratornoye issledovaniye). Abstract on Ph.D. thesis defense: 14.00.18. Moscow: Moscow Scientific Research Institute of Psychiatry, 1968. (In Russ.) p.24.

Hungary

Arato M, O'Connor R, Meltzer HY, Group ZS. A 1-year, double-blind, placebo-controlled trial of ziprasidone 40, 80 and 160 mg/day in chronic schizophrenia: the Ziprasidone Extended Use in Schizophrenia (ZEUS) study. Int Clin Psychopharmacol (2002) 17(5):207-15. doi: 10.1097/00004850-200209000-00001

Balogh N, Egerhazi A, Berecz R, Csukly G. Investigating the state-like and trait-like characters of social cognition in schizophrenia: a short term follow-up study. Schizophr Res (2014) 159(2-3):499-505. doi: 10.1016/j.schres.2014.08.027

Balogh N, Egerhazi A, Berecz R. Neurocognitive changes in patients with schizophrenia during relapse and early remission. European Journal of Psychiatry (2015) 29(3):199-209. doi: 10.4321/s0213-61632015000300004.

Bitter I, Jaeger J, Agdeppa J, Volavka J. Subjective symptoms: part of the negative syndrome of schizophrenia? Psychopharmacol Bull (1989) 25(2):180-4.

Bitter I. (editor). Managing Negative Symptoms of Schizophrenia: Oxford University Press (2020). 144 p.

Bitter I. Definitions and measurement of negative symptoms in schizophrenia. In: Bitter I, editor. Managing Negative Symptoms of Schizophrenia. Oxford University Press (2020). p. 1-18.

Corponi F, Fabbri C, Bitter I, Montgomery S, Vieta E, Kasper S, et al. Novel antipsychotics specificity profile: A clinically oriented review of lurasidone, brexpiprazole, cariprazine and lumateperone. Eur Neuropsychopharmacol (2019) 29(9):971-85. doi: 10.1016/j.euroneuro.2019.06.008

Csukly G, Polgar P, Tombor L, Benkovits J, Rethelyi J. Theory of mind impairments in patients with deficit schizophrenia. Compr Psychiatry (2014) 55(2):349-56. doi: 10.1016/j.comppsych.2013.08.025

Czobor P, Bitter I, Volavka J. Relationship between the Brief Psychiatric Rating Scale and the Scale for the Assessment of Negative Symptoms: a study of their correlation and redundancy. Psychiatry Res (1991) 36(2):129-39. doi: 10.1016/0165-1781(91)90125-9

Czobor P, Bitter I. Pharmacologic treatment of negative symptoms: focus on efficacy. In: Bitter I, editor. Managing Negative Symptoms of Schizophrenia Oxford University Press (2020). p. 67-86.

Farkas M, Polgar P, Kelemen O, Rethelyi J, Bitter I, Myers CE, et al. Associative learning in deficit and nondeficit schizophrenia. Neuroreport (2008) 19(1):55-8. doi: 10.1097/WNR.0b013e3282f2dff6

Galderisi S, Mucci A, Bitter I, Libiger J, Bucci P, Fleischhacker WW, et al. Persistent negative symptoms in first episode patients with schizophrenia: results from the European First Episode Schizophrenia Trial. Eur Neuropsychopharmacol (2013) 23(3):196-204. doi: 10.1016/j.euroneuro.2012.04.019

Galderisi S, Mucci A, Dollfus S, Nordentoft M, Falkai P, Kaiser S, et al. EPA guidance on assessment of negative symptoms in schizophrenia. Eur Psychiatry (2021) 64(1):e23. doi: 10.1192/j.eurpsy.2021.11

Jaeger J, Bitter I, Czobor P, Volavka J. The measurement of subjective experience in schizophrenia: the Subjective Deficit Syndrome Scale. Compr Psychiatry (1990) 31(3):216-26. doi: 10.1016/0010-440x(90)90005-d

Kirkpatrick B, Cohen A, Bitter I, Strauss GP. Primary Negative Symptoms: Refining the Research Target. Schizophr Bull (2021) 47(5):1207-10. doi: 10.1093/schbul/sbab069

Lindenmayer JP, Kahn A. Pszichopatológia. In: Lieberman A, Stroup S, Perkins D, editors. A szkizofrénia tankönyve (The American Psychiatric Publishing textbook of schizophrenia). Budapest: Lélekben Otthon Kft (2006). p. 187-223.

Mucci A, Vignapiano A, Bitter I, Austin SF, Delouche C, Dollfus S, et al. A large European, multicenter, multinational validation study of the Brief Negative Symptom Scale. Eur Neuropsychopharmacol (2019) 29(8):947-59. doi: 10.1016/j.euroneuro.2019.05.006

Nemeth G, Laszlovszky I, Czobor P, Szalai E, Szatmari B, Harsanyi J, et al. Cariprazine versus risperidone monotherapy for treatment of predominant negative symptoms in patients with schizophrenia: a randomised, double-blind, controlled trial. Lancet (2017) 389(10074):1103-13. doi: 10.1016/S0140-6736(17)30060-0

Polgár P, Farkas M, Nagy O, Kelemen O, Réthelyi J, Bitter I, et al. How to find the way out from four rooms? The learning of "chaining" associations may shed light on the neuropsychology of the deficit syndrome of schizophrenia. Schizophr Res (2008) 99(1-3):200-7. doi: 10.1016/j.schres.2007.06.027

Réthelyi JM, Bakker SC, Polgár P, Czobor P, Strengman E, Pásztor PI, Kahn RS, Bitter I. Association study of NRG1, DTNBP1, RGS4, G72/G30, and PIP5K2A with schizophrenia and symptom severity in a Hungarian sample. American Journal of Medical Genetics Part B: Neuropsychiatric Genetics. 2010 Apr;153(3):792-801. doi.org/10.1002/ajmg.b.31049

Vokó Z, Bitter I, Mersich B, Réthelyi J, Molnár A, Pitter JG, et al. Using informative prior based on expert opinion in Bayesian estimation of the transition probability matrix in Markov modelling-an example from the cost-effectiveness analysis of the treatment of patients with predominantly negative symptoms of schizophrenia with cariprazine. Cost Eff Resour Alloc (2020) 18:28. doi: 10.1186/s12962-020-00224-w

Kazakhstan

Adilkhanova KA. Paraphrenic syndrome in various types of schizophrenia. Dissertation Abstract for PhD level. Moscow, 1971. 16p.

Altynbekov KS. Optimization of psychiatric aid for patients with schizophrenia in a hospital setting (clinical and therapeutic, organizational and pharmaco-economic aspects). Dissertation Abstract for PhD level. St. Petersburg, 2017. 43p.

Altynbekov SA, Sayatova GU. Clinical and social factors of the rehabilitation potential of the patients with paranoid schizophrenia. (2019).

Bazarbayeva LE. Forensic psychiatric aspect of socially dangerous actions in patients with schizophrenia (clinical-social, clinical-psychopathological research). Dissertation Abstract for PhD level. Almaty, 2010. 46p.

Dzhamantayeva MS. Hypochondriacal disorders in various clinical schizophrenia variants. Dissertation Abstract for PhD level. Moscow, 1983. 16p.

Gubashev MS. Clinical and social characteristics of patients with schizophrenia who have committed murder. Dissertation Abstract for PhD level. Almaty, 1999. 30p.

Izmailova NT. Schizophrenia complicated by the use of hashish. Dissertation Abstract for PhD level. Tashkent, 1998. 48p.

Kokshinova OY. Clinical and psychopathological characteristics of deviant behavior of patients with schizophrenia during the period of compulsory treatment. Dissertation Abstract for PhD level. Almaty, 2010. 26p.

Lyubchenko LY. Clinical and psychopathological picture and oxidative metabolism in continuous and paroxysmal progressive schizophrenia in women. Dissertation Abstract for PhD level. Almaty, 2002. 25p.

Malysheva NV. Internal picture of the disease in patients with schizophrenia in remission. Dissertation Abstract for PhD level. Almaty, 2001. 20p.

Nurkatov EM. Phenomenological features of the Kandinsky-Clérambault syndrome in schizophrenic patients with different cultural and educational levels. Dissertation Abstract for PhD level. Almaty, 2003. 25.

Raspopova NI. Mechanisms of formation, clinical features and prevention of suicidal behavior in patients with mental disorders. Dissertation Abstract for PhD level. Almaty, 2012. 37p.

Raspopova NI. Suicidal behavior of mentally ill patients Clinical and epidemiological study. Palmarium Academic Publishing, 2013. 346p. http://bnd.b-nb.de.

Raspopova NI, Tolstikova AYu. Comparative analysis of psychopathological mechanisms of homicides and suicides in patients with schizophrenia. Journal of Suicidology (2016) 22(1): 40-5.

Raspopova NI. The dynamics of the frequency of suicides in the Republic of Kazakhstan. Suicides in Russia and Europe, edited by B.S. Position. Moscow, 2016. p. 73-100.

Raspopova N.I., Dzhamantaeva M.Sh. The role of ethnocultural factors in the formation of suicidal behavior in persons with mental disorders in the Republic of Kazakhstan. Journal of Medical News of Georgia (2018) 11(284):66-70.

Raspopova NI, Dzhamantaeva MS, Bastasova UA, Suleimenova AA, Boyko VS, Logacheva NN. On the issue of differential diagnosis of pseudoneurotic schizophrenia and neuroses. Bulletin of the Kazakh National Medical University (2020) 4:253–8.

Raspopova NI, Eshimbetova SZ. Modern possibilities of psychopharmacotherapy of negative disorders in the clinical picture of schizophrenia. Medicine (2020) 7-8(217-218):53-61.

Raspopova NI, Bastasova UA, Eshimbetova SZ. Negative disorders in the clinical picture of schizophrenia. Study guide: Lambert Academic Publishing (2021). 60 p.

Raspopova NI, Dzhamantaeva MS, Bastasova UA. Negative disorders as the main differential diagnostic sign of schizophrenia. Psychiatry, Psychotherapy and Narcology (2021) 1:37-41.

Saduakasova KZ. Clinical and genetic characteristics of schizophrenia in children and adolescents in the Kazakh population. Dissertation Abstract for PhD level. Almaty, 2006. 30p.

Lithuania

Brady RO, Gonsalvez I, Lee I, Öngür D, Seidman LJ, Schmahmann JD, et al. Cerebellar-Prefrontal Network Connectivity and Negative Symptoms in Schizophrenia. Am J Psychiatry (2019) 176(7):512-20. doi: 10.1176/appi.ajp.2018.18040429

Correll CU, Schooler NR. Negative Symptoms in Schizophrenia: A Review and Clinical Guide for Recognition, Assessment, and Treatment. Neuropsychiatr Dis Treat (2020) 16:519-34. doi: 10.2147/NDT.S225643

Galderisi S, Mucci A, Buchanan RW, Arango C. Negative symptoms of schizophrenia: new developments and unanswered research questions. Lancet Psychiatry (2018) 5(8):664-77. doi: 10.1016/S2215-0366(18)30050-6

Kumar N, Vishnubhatla S, Wadhawan AN, Minhas S, Gupta P. A randomized, double blind, sham-controlled trial of repetitive transcranial magnetic stimulation (rTMS) in the treatment of negative symptoms in schizophrenia. Brain Stimul (2020) 13(3):840-9. doi: 10.1016/j.brs.2020.02.016

Montvidas J, Adomaitiene V, Leskauskas D, Dollfus S. Validation of the lithuanian version of the self-evaluation of negative symptoms scale (SNS). Nord J Psychiatry (2021) 75(5):351-5. doi: 10.1080/08039488.2020.1862295

Thonon B, Van Aubel E, Lafit G, Della Libera C, Laroi F. Idiographic analyses of motivation and related processes in participants with schizophrenia following a therapeutic intervention for negative symptoms. BMC Psychiatry (2020) 20(1):464. doi: 10.1186/s12888-020-02824-5

Latvia

Marder SR, Galderisi S. The current conceptualization of negative symptoms in schizophrenia. World Psychiatry (2017) 16(1):14-24. doi: 10.1002/wps.20385

World Health Organization. (2019). International Statistical Classification of Diseases and Related Health Problems 10th Revision (ICD-10)-WHO Version for; 2019-covid-expanded. https://icd.who.int/browse10/2019/en#/F20-F29 [Accessed November 8, 2021].

Moldova

Nacu A, Chihai J, Coșciug I, Deliv I, Garaz G, Oprea V, et al. Manual de psihiatrie (Manual of psychiatry): Topography Bons Offices (2021). 647 p.

Protocolul Clinic Național nr. 9 al MSMPS – Schizofrenia, Primul episode psihotoc (National Clinical Guideline Schizophrenia, First Psychotic episode - nr. 9 from 2020 – Ministry of Health, Labor, Social Protection) http://sanatatemintala.md/ro/legislatie/legislatia-nationala

Poland

Bienkowski P, Samochowiec J, Pelka-Wysiecka J, Grzywacz A, Skibinska M, Jasiewicz A, et al. Functional polymorphism of matrix metalloproteinase-9 (MMP9) gene is not associated with schizophrenia and with its deficit subtype. Pharmacol Rep (2015) 67(3):442-5. doi: 10.1016/j.pharep.2014.11.007

Michalczyk A, Pelka-Wysiecka J, Kucharska-Mazur J, Wronski M, Misiak B, Samochowiec J. Association between DRD2 and ANKK1 polymorphisms with the deficit syndrome in schizophrenia. Ann Gen Psychiatry (2020) 19:39. doi: 10.1186/s12991-020-00289-0

Misiak B, Frydecka D, Beszlej JA, Moustafa AA, Tybura P, Kucharska-Mazur J, et al. Effects of antipsychotics on insight in schizophrenia: results from independent samples of first-episode and acutely relapsed patients. Int Clin Psychopharmacol (2016) 31(4):185-91. doi: 10.1097/YIC.0000000000000120

Misiak B, Bienkowski P, Samochowiec J. Cariprazine - a novel antipsychotic drug and its place in the treatment of schizophrenia. Psychiatr Pol (2018) 52(6):971-81. doi: 10.12740/PP/OnlineFirst/80710

Piotrowski P, Kotowicz K, Rymaszewska J, Beszlej JA, Plichta P, Samochowiec J, et al. Allostatic load index and its clinical correlates at various stages of psychosis. Schizophr Res (2019) 210:73-80. doi: 10.1016/j.schres.2019.06.009

Pełka-Wysiecka J, Wroński M, Jasiewicz A, Grzywacz A, Tybura P, Kucharska-Mazur J, et al. BDNF rs 6265 polymorphism and COMT rs 4680 polymorphism in deficit schizophrenia in Polish sample. Pharmacol Rep (2013) 65(5):1185-93. doi: 10.1016/s1734-1140(13)71476-2

Podwalski P, Tyburski E, Szczygiel K, Waszczuk K, Rek-Owodzin K, Mak M, et al. White Matter Integrity of the Corpus Callosum and Psychopathological Dimensions in Deficit and Non-Deficit Schizophrenia Patients. J Clin Med (2021) 10(11). doi: 10.3390/jcm10112225

Samochowiec J, Szulc A, Bieńkowski P, Dudek D, Gałecki P, Heitzman J, et al. Polish Psychiatric Association consensus statement on non-pharmacological methods in the treatment of negative symptoms of schizophrenia. Psychiatria Polska (2021) 55(4):719-42. doi: 10.12740/PP/OnlineFirst/135527.

Samochowiec J, Tyburski E, Blazej M. Schizofrenia u mężczyzn (Schizophrenia in Men). Warszawa: Medical Education (2020).

Szulc A, Samochowiec J, Galecki P, Wojnar M, Heitzman J, Dudek D. Recommendations for the treatment of schizophrenia with negative symptoms. Standards of pharmacotherapy by the Polish Psychiatric Association (Polskie Towarzystwo Psychiatryczne), part 1. Psychiatr Pol (2019) 53(3):497-524. doi: 10.12740/PP/OnlineFirst/100698

Szulc A, Dudek D, Samochowiec J, Wojnar M, Heitzman J, Galecki P. Recommendations for the treatment of schizophrenia with negative symptoms. Standards of pharmacotherapy by the Polish Psychiatric Association (Polskie Towarzystwo Psychiatryczne), part 2. Psychiatr Pol (2019) 53(3):525-40. doi: 10.12740/PP/OnlineFirst/100697

Szulc A, Samochowiec J, Marciniak A. Schizofrenia z objawami negatywnymi. Obciążenie chorobą pacjentów i ich bliskich (Schizophrenia with negative symptoms. The burden of the disease on patients and their families). Warszawa: PZWL (2019).

Tyburski E, Mak M, Samochowiec A, Plichta P, Bielecki M, Rek-Owodzin K, et al. The relationship between cingulum bundle integrity and different aspects of executive functions in chronic schizophrenia. Prog Neuropsychopharmacol Biol Psychiatry (2020) 102:109955. doi: 10.1016/j.pnpbp.2020.109955

Tyburski E, Mak M, Sokolowski A, Starkowska A, Karabanowicz E, Kerestey M, et al. Executive Dysfunctions in Schizophrenia: A Critical Review of Traditional, Ecological, and Virtual Reality Assessments. J Clin Med (2021) 10(13). doi: 10.3390/jcm10132782

Wichniak A, Siwek M, Rymaszewska J, Janas-Kozik M, Wolańczyk T, Bieńkowski T, Dudek D, Heitzman J, Szulc A, Samochowiec J. Stanowisko grupy roboczej Polskiego Towarzystwa Psychiatrycznego na temat stosowania częściowych agonistów receptorów dopaminowych D2/D3 w leczeniu zaburzeń psychicznych. (The position statement of the Working Group of the Polish Psychiatric Association on the use of D2/D3 dopamine receptor partial agonists in special populations). in print Psychiatria Polska (2021).

Wójciak P, Rybakowski, J. Obraz kliniczny, patogeneza i ocena psychometryczna objawów negatywnych schizofrenii. (Clinical picture, pathogenesis and psychometric assessment of negative symptoms of schizophrenia). Psychiatria Polska. (2018) 52(2):185–97.

Romania

Căpățînă OO, Micluția IV. Negative Symptoms of Schizophrenia: From Kraepelin to DSM 5. Romanian Journal of Psychiatry (2015) 17(3):48-50.

Căpățînă OO, Miclutia IV. Are negative symptoms in schizophrenia a distinct therapeutic target? Clujul Med (2018) 91(1):58-64. doi: 10.15386/cjmed-864

Căpățînă OO, Micluția I. Internalized stigma as a predictor of quality of life in schizophrenia. J Evid Based Psychother (2018) 19:35-53. doi: 10.24193/jebp.2018.2.13.

Căpățînă OO, Campean A, Toma A. Global functioning and quality of life in primary versus secondary negative symptoms in schizophrenia. 26th European Congress of Psychiatry; 2018: European Psychiatry (2018). p. S327-S. PW0538.

Căpățînă OO, Miclutia I. Course of negative symptoms subdomains in schizophrenia: A one year follow-up study. 26th European Congress of Psychiatry; 2018: European Psychiatry (2018). p. S348-S. PW0859.

Căpățînă OO, Androne B, Stanculete MF, editors. Secondary negative symptoms in schizophrenia. 27th European Congress of Psychiatry; 2019 2019. E-PP1053.

Căpățînă OO. Simptomele negative în schizofrenie: aspecte clinicoevolutive (Negative symptoms in schizophrenia: clinical and evaluative aspects). PhD Dissertation. Iuliu Hatieganu University of Medicine and Pharmacy, Cluj-Napoca, 2019 (In Romanian)

Căpățînă OO, Miclutia IV. Internalized stigma, negative symptoms and global functioning in schizophrenia. European Psychiatry (2020) 33(S1):S574-S5. doi: 10.1016/j.eurpsy.2016.01.2127.

Căpățînă OO, Miclutia IV, toma A. Relationship between cognition and primary negative symptoms sub-domains in schizophrenia. European Psychiatry (2020) 41(S1):S188-S. doi: 10.1016/j.eurpsy.2017.01.2112.

Căpățînă O, Stanculete MF, Miclutia I. Behavioral outputs of negative symptom domains of schizophrenia. Exp Ther Med (2021) 22(2):805. doi: 10.3892/etm.2021.10237

Căpățînă OO, Miclutia IV, Fadgyas-Stanculete M. Current perspectives in treating negative symptoms of schizophrenia: A narrative review (Review). Exp Ther Med (2021) 21(3):276. doi: 10.3892/etm.2021.9707

Deac C, Macrea R, Popescu CA, Valentina I. Discrimination learning and set-shifting in schizophrenia: associations with negative symptoms. AMT, 2014, 2 (1), 173-175.

Fadgyas-Stanculete M, Capatina O. The Many Faces of Negative Symptoms in Schizophrenia. In: Fukao K, editor. Psychosis - Phenomenology, Psychopathology and Pathophysiology [Working Title]. (2021).

Micluţia, I., Macrea, R., Ionaşcu, R. Schizophrenia with negative symptoms, Bull. De l'Union Medicale Balkanique,153, 1995.

Russia

Ivanov M.V., Neznanov N.G. Negativnye I kognitivnye rasstroistva pri endogennyh psihosah (Negative and cognitive disorders in endogenous psychoses). Saint Petersburg. (2008). 287 p. (In Russ.).

Juckel G, de Bartolomeis A, Gorwood P, Mosolov S, Pani L, Rossi A, et al. Towards a framework for treatment effectiveness in schizophrenia. Neuropsychiatr Dis Treat (2014) 10:1867-78. doi: 10.2147/NDT.S61672

Mosolov S.N. Shkaly psihometricheskoi otsenki simptomatiki shizofrenii I kontseptsia positivnyh I negativnyh rasstroystv (Psychometric scales of schizophrenia symptoms and the concept of positive and negative disorders). Moscow. Novyi Tsvet (2001). 238 p. (In Russ.).

Mosolov SN, Potapov AV, Ushakov UV. Remission in schizophrenia: results of cross-sectional with 6-month follow-up period and 1-year observational therapeutic studies in an outpatient population. Ann Gen Psychiatry (2012) 11(1):1. doi: 10.1186/1744-859X-11-1

Mosolov SN, Potapov AV, Ushakov UV, Shafarenko AA, Kostyukova AB. Design and validation of standardized clinical and functional remission criteria in schizophrenia. Neuropsychiatr Dis Treat (2014) 10:167-81. doi: 10.2147/NDT.S46799

Smulevich AB, Iastrebov VS, Izmailova LC. Tipologia defektnyh sostoyanyi s sindromom monotonnoyi aktivnosti u bol'nyh shizofreniei (problema pozdnih remissyi). (Typology of defective states with a syndrome of monotonous activity in schizophrenic patients (problem of late remissions)). Zh Nevropatol Psikhiatr Im S S Korsakova (1976) 76(9):1372-9. (In Russ.).

Smulevich AB, Lobanova VM, Voronova EI. Negativnye rasstroistva: istoria I klinicheskie kontseptsii (prenozologicheskyi period). (Negative disorders: history of clinical concepts (prenosological studies)). Zh Nevrol Psikhiatr Im S S Korsakova (2021) 121(1):84-91. doi: 10.17116/jnevro202112101184 (In Russ.).

Snezhnevsky A.V. O nozologicheskoy spetsifichnosti psihopatologicheskih sindromov (About the nosological specificity of psychopathological syndromes). Zhurnal Nevrologii I Psihiatrii S.S. Korsakova (1960) 60(1):91-110 (In Russ.).

Snezhnevsky AV. Shizofrenia (multidistsiplinarnoe issledovanie) (Schizoprenia (Multidisciplnaty Study)). Moscow: Medicine (1972). (In Russ).

Tiganov AS, Snezhnevsky AV, Orlovskaya DD. Obschaya psihiatria. Rukovodstvo po psychiatrii. Tom 1 (General psychiatry. Handbook of Psychiatry. Vol.1). Moscow: Meditsina (1999). 784 p. (In Russ.).

Serbia

Jašović-Gašič M, Maric N. Shizofrenija (Schizophrenia). In: Jašovič-Gašić M, Lečić-Toševski D, editors. Psihijatrija, udžbenik za studente medicine. Belgrade: CIBID Medicinski fakultet (2014) (in Serbian).

Lečić-Tosevski D. Nacionalni vodič dobre kliničke prakse za dijagnostikovanje i lečenje shizofrenije. Republička stručna komisija za izradu i implementaciju vodiča u kliničkoj praksi (National guide - good clinical practice for the diagnosis and treatment of schizophrenia. Republic Expert Commission for the Development and Implementation of Guides in Clinical Practice). Belgrade: Ministarstvo zdravlja Republike Srbije (2013) (in Serbian).

Marić N, Miljević Č, Mihaljević M. Psihotični poremećaji (Psychotic disorders). In: Jovanović A, editor. Psihijatrija, udžbenik za studente medicine (Psychiatry, a textbook for medical students). Belgrade: CIBID Medicinski fakultet – in press (In Serbian).

Ristic I, Jerotic S, Zebic M, Savic B, Vukovic V, Russo M, et al. Factorial Structure of the Serbian Version of the Clinical Assessment Interview for Negative Symptoms - Evidence for Three Factors of Negative Symptoms. Front Psychol (2020) 11:570356. doi: 10.3389/fpsyg.2020.570356

Slovakia

Cerveri G, Gesi C, Mencacci C. Pharmacological treatment of negative symptoms in schizophrenia: update and proposal of a clinical algorithm. Neuropsychiatr Dis Treat (2019) 15:1525-35. doi: 10.2147/NDT.S201726

Dragašek J, Vančová Z. Súčasné možnosti a perspektíva liečby negatívnych príznakov schizofrénie. (Current options and perspectives for the treatment of negative symptoms associated with schizophrenia). Psychiatr prax (2018) 19(2):57-62.

Masopust J, Mohr P, Kopeček M. Antipsychotika v léčbě predominantních negativních příznaků schizofrenie: aktualizace doporučených postupů. (Antipsychotics in treatment of predominant negative symptoms in schizophrenia: an update of guidelines). Psychiatrie (2020) 24(1):40-3.

Masopust J, Kopeček M, Protopopová D. DOPORUČENÉ POSTUPY PSYCHIATRICKÉ PÉČE 2020: Stabilizační a udržovací léčba schizofrenie. (Stabilizing and maintenance treatment of schizophrenia. Guidelines of psychiatric care 2018 (update 2020)). Psychiatrie (2020).

Pečeňák, J. Schizofrénia – koncepty, symptómy, diagnostické kritériá. (Schizophrenia - concepts, symptoms, diagnostic criteria). Bratislava: Univerzita Komenského (2014).

Vančová Z, Dragašek J. Antipsychotiká v liečbe negatívnych príznakov schizofrénie – čo hovoria aktualizované odporúčané postupy? (Antipsychotics in the treatment of predominant negative symptoms in schizophrenia - What are the updated guidelines telling us?). Psychiatria Pre Prax (2020) 21(4):162–5.

Zelman M, Pečeňák J, Breznoščáková D, Kubašovská K. Komplexný manažment pacienta so schizofréniou (Complex management of patients with schizophrenia). Bratislava: Ministry of Health of Slovak Republic (2019).

Slovenia

Pregelj P, Kores-Plesničar B, Tomori M, Zalar B, Ziherl S, editors. Psihiatrija (Psychiatry). Ljubljana: Psihiatrična klinika (2013).

Tavcar R. Metodološki in klinični vidiki prognoze shizofrenije. (Methodologic and clinical aspects of the prognosis of schizophrenia). Med Razgl (1994) 33: 533–40.

Ukraine

Khomitskyi ME. Interrelation of clinical-anamnestic, medical-biological and neurocognitive characteristics of maladaptation in remission as a component of pathopersonological transformations in patients with schizoaffective disorder. Ukrains'kyi Visnyk Psykhonevrolohii 28(1 (102)):68-74.

Kolyadko S, Kalenska G. Peculiarities of Art Therapy Using in Treatment of Patients with Paranoid Schizophrenia. Ukrains'kyi Visnyk Psykhonevrolohii (2017) 25(1):51-6.

Kryshtal V. Evaluation of the eff ectiveness of the psychoeducation system in patients with schizophrenia. Ukrains'kyi Visnyk Psykhonevrolohii (2017) 25(1):56-9.

Maruta NO, Kalenska GY, Maliuta LV. Clinical-psychopathological and pathopsychological peculiarities of depressive disorders in patients with schizophrenia. ScienceRise (2016) 4(21):9-15.

Maruta NO, Bilous VS. Prodromal period of psychosis: clinical-psychopathological and pathopsychological patterns of formation, diagnostic criteria and principles of prevention. Bulletin of scientific research (2017) 4:81-5.

Maruta NO, Chaban OS. Choice of effective antipsychotic therapy to combat positive and negative symptoms of schizophrenia. Health of Ukraine (2020) 2:46-7.

Maruta NO, Chaban OS. Choosing an effective antipsychotic therapy to combat the positive and negative symptoms of schizophrenia. Health of Ukraine (2020) 2:46-7.

Moseyko AV. Positive and negative features of psychopathological symptoms atatypical variants of simple-type schizophrenia. Journal of Education, Health and Sport (2017) 7(6):597-603. doi: 10.5281/zenodo.829972

Putyatin GG. Diagnostic Criteria and Clinical Characteristics of Hospitalism Syndrome in Patients with Schizophrenia. Journal of Psychiatry and Medical Psychology (2008) 3(20):16-21.

Skrypnikov AM, Kydon’ PV. Etiology and pathogenesis of schizophrenia: the current state of the problem: ASMI (2019). 51 p.

Syropyatov OG. Modern treatment of schizophrenia. Neuronews (2007) 2(3).

Yurieva LN. Schizophrenia: A Clinical Guide for Physicians. Kiev (2010). 244 p.

frontiers
